# The contexts of heavy drinking: A systematic review of the combinations of context-related factors associated with heavy drinking occasions

**DOI:** 10.1371/journal.pone.0218465

**Published:** 2019-07-10

**Authors:** Oliver Stanesby, Florian Labhart, Paul Dietze, Cassandra J. C. Wright, Emmanuel Kuntsche

**Affiliations:** 1 Centre for Alcohol Policy Research, La Trobe University, Melbourne, Australia; 2 Idiap Research Institute, Martigny, Switzerland; 3 Addiction Switzerland, Research Institute, Lausanne, Switzerland; 4 Burnet Institute, Melbourne, Australia; 5 School of Public Health and Preventive Medicine, Monash University, Melbourne, Australia; 6 Behavioural Science Institute, Radboud University, Nijmegen, The Netherlands; University of the Witwatersrand, SOUTH AFRICA

## Abstract

**Background:**

The amount of alcohol consumed during an occasion can be influenced by physical and social attributes of the setting, characteristics and state of individuals, and the interactions of these components. This systematic review identifies and describes the specific combinations and sequences of context-related factors that are associated with heavy drinking occasions.

**Materials and methods:**

We conducted a systematic literature search of MEDLINE, Embase and the Cumulative Index to Nursing and Allied Health Literature (CINAHL) databases. Eligible articles were event-level and event-based studies that quantitatively analysed associations of sequences or combinations of context-related factors with event-level alcohol consumption. We extracted information on study design, sample, variables, effect estimates and analytical methods. We compiled a list of combinations and sequences associated with heavier drinking (i.e., ‘risky contexts’) and with lighter drinking (‘protective contexts’). The review protocol was registered with PROSPERO (registration number: CRD42018089500).

**Results:**

We screened 1902 retrieved records and identified a final sample of 65 eligible studies. Daily mood, day of week, location and drinking group characteristics are important drivers of whether an individual engages in a heavy drinking occasion. The direction and magnitude of some associations differed by gender, age, personality and motives, such that in particular social or physical contexts, some people may feel compelled to drink more while others are compelled to drink less. Very few sequences of factors were reported as being associated with event-level alcohol consumption.

**Conclusions:**

Contexts or factors are experienced in specific sequences that shape the broader drinking context and influence drinking behaviours and consequences but are under-studied. Event-level studies such as those using ecological momentary assessment can harness new technologies for data collection and analysis to improve understandings of why people engage in heavy drinking. Continued event-level research will facilitate public health interventions and policies that reduce heavy drinking and alcohol-related harms.

## 1. Introduction

Heavy drinking can result in harm at the individual, familial, community and societal levels [1–4]. Drinking tends to occur in specific contexts. The social and physical characteristics of contexts as well as the characteristics and state of the individual can influence whether they engage in heavy drinking and whether alcohol-related consequences are experienced [5–10]. Contextual factors may combine or co-occur during a drinking occasion in a specific manner that shapes the broader drinking context and influences an individuals’ drinking. A comprehensive review of the literature is needed to compile and summarise the specific contexts associated with heavy drinking patterns to reveal opportunities for effective environmental approaches to reduce alcohol-related harms due to heavy drinking.

### 1.1. Heavy drinking

Heavy drinking patterns (commonly termed ‘binge’, ‘risky single occasion’, ‘heavy episodic’ or ‘short-term risky’ drinking) involve consuming a relatively high amount of alcohol in a relatively short period of time [3, 11, 12]. Heavy drinking is most common on Friday and Saturday nights when young people go out and have few work or study responsibilities the following day [13, 14]. Short term consequences of heavy drinking include blackout, memory loss, nausea, vomiting, hangovers, alcohol poisoning, unintended and/or unprotected sexual activity, injury, traffic accidents, and death [2, 3, 11, 15–17]. Those who engage in heavy drinking may also be more likely to harm others, for example via vandalization, inter-personal violence and aggression, and traffic accidents due to drink-driving [3, 4, 18–22]. While heavy drinking is typically discussed in terms of immediate harms, consequences can also be long-term (e.g., permanent disability from an injury sustained during a traffic accident). Furthermore, there is evidence indicating heavy drinking in early life is linked to numerous long-term negative consequences [3, 11, 17, 23, 24].

Event-level alcohol consumption refers to an individuals’ drinking pattern during a given occasion. An occasion typically refers to a day or evening, but may be more specific (e.g., during a visit to a venue). Event-level alcohol consumption is distinct from measures of alcohol consumption across longer time-periods (e.g., average daily alcohol consumption in the last 12 months, usual alcohol consumption per drinking occasion in last week). Unless otherwise stated, hereafter ‘drinking’ refers to event-level alcohol consumption, ‘heavy’ or ‘heavier’ drinking refers to higher event-level alcohol consumption, and ‘light’ or lighter’ drinking refers to lower event-level alcohol consumption.

### 1.2. Drinking occurs in complex contexts that influence drinking behaviour

According to a social ecological perspective of human behaviour [5, 6, 8–10], the immediate drinking context can be characterised by the physical and social attributes of the setting, the characteristics and state of individuals, and the interactions of these components. This model implies that contextual factors influence whether an individual engages in heavy drinking and whether alcohol-related consequences are experienced. Further, associations between individual-level factors (e.g., gender, age, personality and motives) and heavy drinking may be altered by contextual factors (e.g., time, place, occasion and presence of others).

### 1.3. The broader drinking context: a sequence of immediate drinking contexts

**Fig 1:**
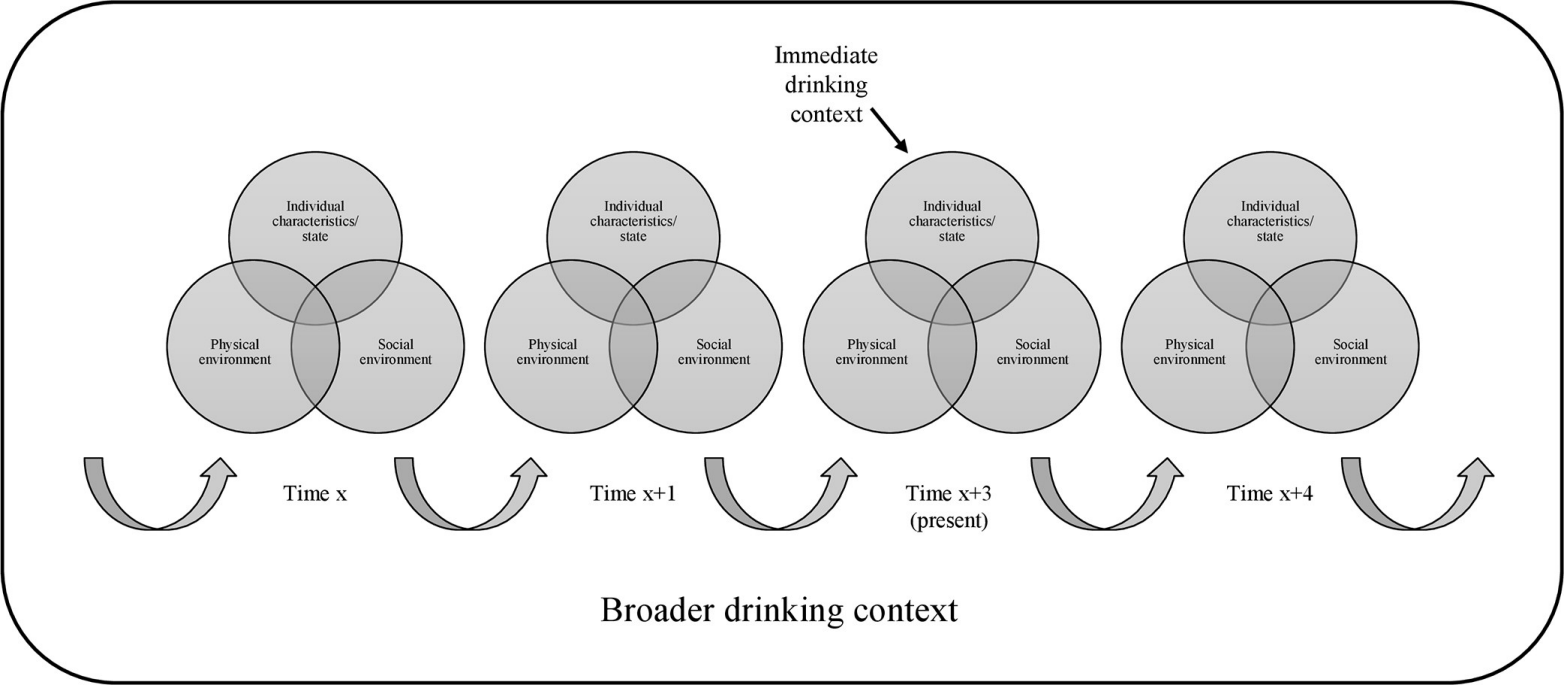
The broader drinking context: Comprising a sequence of immediate drinking contexts which are described by combinations of factors related to the characteristics and state of individuals, the physical environment and the social environment ^1^. ^1^ As per a social ecological perspective of human behaviour [5, 6, 8–10].

Previous studies have focused on the independent effects of factors on drinking occasions and consequences (e.g., [25–28]. However, in the real world, factors may combine or co-occur during a drinking occasion in a specific sequential manner that shapes the broader drinking context and influences an individuals’ drinking. Fig 1 provides a visual depiction of the broader drinking context. As shown, the broader drinking context comprises a sequence of immediate drinking contexts that are described by combinations and sequences of factors related to the characteristics and state of individuals, the physical environment and the social environment. Pre-drinking or pre-loading provides an example of a risky sequential combination of context-related factors—the act of drinking alcohol, usually at a domestic residence, prior to attending a social event, typically at a bar or nightclub [29, 30]. Occasions that include pre-drinking are associated with heavier drinking than those that do not [29, 31–34]. Moving between several outlets (e.g., pub crawling) provides another example of a risky combination of sequential factors.

Individual-level factors may also moderate associations between sequences of factors and drinking; for example, the positive association between pre-drinking and likelihood of heavy drinking is stronger among men with lower conformity drinking motives than men with higher conformity motives [35].

The complexity of the broader drinking context (Fig 1) means that it is imperative for research to consider the interplay (i.e., interaction or combined effect) of multiple factors relating to the context and the individual (rather than simply estimating the independent effect of one factor), as well as the specific sequence of these factors. This is necessary to comprehensively explain associations between drinking contexts and heavy drinking and alcohol-related harms.

### 1.4. Studying drinking contexts and event-level associations

Two main types of studies have been used to investigate simultaneous and/or prospective relationships of context-related factors and individual-level factors with drinking. The first is *event-level* studies, which collect data during the event(s) using methods such as ecological momentary assessment (EMA). The second is *event-based* studies, which collect data retrospectively about an event (e.g., retrospective survey) or events (e.g., timeline follow back; TLFB). In theory, both designs allow the exploration of event-level associations between specific contexts and heavy drinking. However, their ability to comprehensively describe contexts and explore event-level associations between contexts and drinking varies.

Some event-level and event-based studies have identified specific combinations of factors related to drinking contexts and/or individuals that are linked to heavier drinking occasions among adolescents and young adults. For example, Thrul and Kuntsche found that the positive association between drinking with friends and heavier drinking was stronger among males than females [36]. Lau-Barraco and colleagues report that drinking occasions were particularly heavy on weekend days for those with higher social alcohol expectancies [37]. Another study reported that occasions spent in public locations with few intoxicated people are associated with lighter drinking [38].

### 1.5. The need for a review of the literature on the contexts of heavy drinking

To our knowledge, no review has comprehensively summarised the evidence on the specific contexts that are associated with heavy drinking. Further, we found no studies that comprehensively reviewed and discussed the design and methodology of these studies. A comprehensive review of the literature is needed to compile and summarise the specific contexts associated with heavy drinking patterns.

To address these gaps, we conducted a systematic review which:

1. Summarises the immediate contexts (described by specific combinations of context-related and individual-level factors) and broader contexts (described by specific sequences of immediate contexts) that are associated with heavier drinking occasions. It provides a more comprehensive understanding of how contexts influence drinking, highlights gaps in understanding of contexts influencing heavy drinking and suggests opportunities for effective environmental approaches to reduce alcohol-related harms due to heavy drinking; and
2. Summarises, critiques and proposes ways to improve the design and analytical methodology of the scientific literature that investigates event-level associations between contexts and drinking.

## 2. Materials and methods

### 2.1. Study identification, eligibility and screening

A systematic review of English articles using MEDLINE, Embase and the Cumulative Index to Nursing and Allied Health Literature (CINAHL) databases was conducted to identify eligible articles. The review protocol was registered with PROSPERO (registration number: CRD42018089500). The review was conducted in line with the PRISMA Statement [39] (see S1 Table for completed PRISMA checklist). Given the complexity of drinking contexts, this review targeted studies that provided descriptions of drinking contexts via sequences or combinations of multiple factors related to the context. Articles eligible for inclusion in data extraction and synthesis were those which: (a) included a quantitative analysis of event-level data; (b) estimated associations of sequences or combinations of two or more factors (at least one context-level variable) with event-level alcohol consumption of the individual; and (c) reported a combination or sequence that was directly associated with significantly increased (‘risky’) or decreased (‘protective’) event-level alcohol consumption, and (d) sampled from broadly Western countries (this criterion was added after the full-text screening phase and prior to data extraction because the majority of eligible studies sampled from Western countries). To maximise the comparability of the findings with general populations, studies with samples comprised only of those with alcohol use disorder (for example) were ineligible. Associations eligible for extraction (Table 1) were those which:

- statistically tested for differences in effect between groups of respondents described by combinations of factors (e.g., via t-test, odds ratio, beta coefficient, interaction), or it was possible to conservatively test via 95% confidence intervals, or reported the level of endorsement of responses in latent class analysis; and
- observed a statistically significant difference (increase or decrease) in the outcome at the 95% confidence level (e.g., p < 0.05 or, when p was not reported, 95% confidence intervals that do not include null or do not overlap), or heavy or lighter drinking endorsed by the majority of a class (>0.50 probability) in latent class analysis.

**Table 1:** Criteria for judging eligibility of retrieved articles for inclusion in data extraction and synthesis.

Design	Quantitative analysis of event-level or event-based data (single or multiple events) obtained in a natural, relatively-normal or generalisable setting
Sample	Human subjects, living in broadly Western countries ^1^, and that are broadly generalisable in terms of socio-demographics and health to general populations
Outcome	Quantitatively-measured event-level alcohol consumption or intoxication level of the individual ^2^
Predictors	Combination or sequence of two or more context-level (includes event-level or event-based) or individual-level factors ^3^ (must be in combination with at least one context-level variable–e.g., via interaction) ^4^^,^^5^
Association	Combination, interaction or sequence of individual-level or context-level factors (predictors) directly associated with increased (‘risky’) or decreased (‘protective’) level of the outcome ^6^^,^^7^
Format	Peer-reviewed scientific original research article using empirical data
Language	Title and abstract in English language in databases searched.

^1^ According to ethnicity, religion and culture (e.g., Christian, European heritage, assimilable to Western culture)

^2^ Event-level alcohol consumption refers to an individuals’ drinking pattern during a given occasion. An occasion typically refers to a day or evening, but may be more specific (e.g., during a visit to a venue).

^3^ Individual-level factors are variables that vary between individuals (not within individuals) and may therefore include individual characteristics (e.g., gender) or typical context (e.g., usual number of licensed venues visited on Saturday nights)

^4^ Thus, an eligible combination may include only factors related to the individual if one of the factors is a context-level variable, therefore describing the state of an individual in-the-event

^5^ Combination, interaction or sequence must not include event-level alcohol consumption, (the outcome)

^6^ Compared to the reference categories of categorical variables and/or the lower values of continuous variables (unless inverted or transformed), as derived by the authors

^7^ Statistically significant difference at the 95% confidence level (P < 0.05 or 95% confidence intervals that do not include null or do not overlap; null associations not extracted), or a class with multiple factors endorsed by the majority of the class (>0.50 probability) had higher event-level alcohol consumption than other class(es) in latent class analysis.

Where studies presented both bivariate and multivariate estimates of a given association, only the effect estimates that are adjusted for potential confounding variables were extracted. For practical and conceptual reasons, qualitative studies and associations of factors with alcohol-related harms (but not event-level alcohol consumption) were not eligible for inclusion in this review. Further details about the eligibility criteria are described in Table 1.

The search strategy was designed in direct consultation with La Trobe University library staff with expertise in conducting systematic review literature searches. To ensure the search was highly sensitive to retrieving eligible records, we used a detailed list of search terms that describe alcohol drinking, event-level or event-based study design, and combinations, interactions or sequences. The search was conducted on the 29^th^ of January 2018. A copy of the full search strategy is provided in S2 Table. Additional relevant articles were identified by contacting experts in this topic of research who suggested articles which were then screened for eligibility.

**Fig 2:**
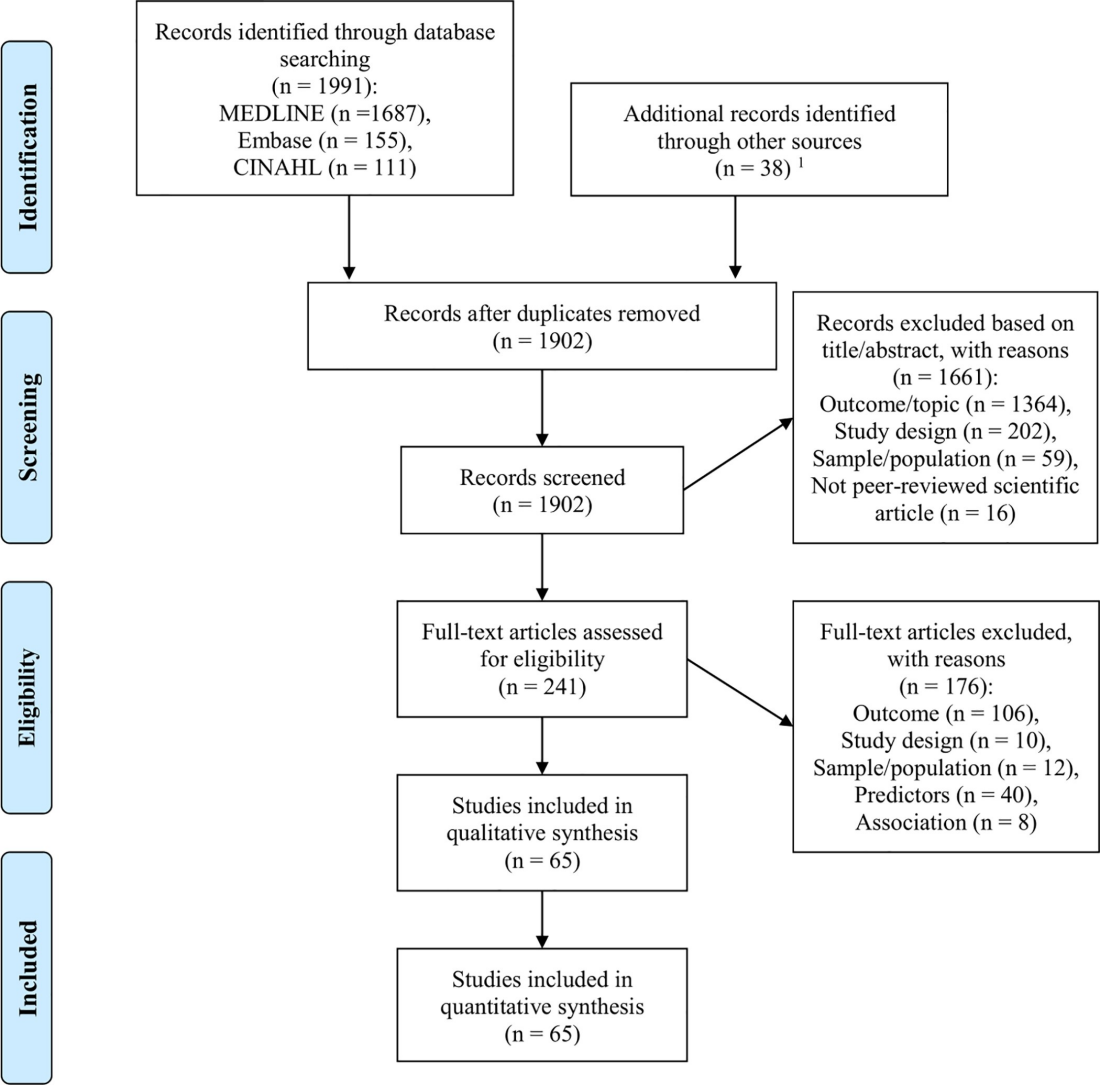
Flow diagram of identification of eligible articles for review. ^1^ Of these, 15 were duplicates, 12 were excluded based on full text and 11 were included in quantitative synthesis; Records eligible for full text screening with no full text published in English were translated to English then screened; Date searched: 29 January 2018; Fig 2 adapted from: [39].

The process for screening records for eligibility is described in Fig 2. Titles and abstracts, then full texts of all retrieved records were screened by an independent researcher (OS) using the criteria described in Table 1. Records that OS was initially unsure whether to include or exclude were screened for eligibility by a second researcher (FL, CW or AP), and differences in opinions of eligibility were resolved via the majority opinion of a group of five researchers (OS, FL, CW, EK and AP).

### 2.2. Data extraction and synthesis

The following information was extracted by OS from the full text of each article included for review:

1. Type of study design: e.g., EMA, daily diary study, TLFB survey, intercept survey, retrospective survey.
2. Sample description: e.g., N; adult, student, patron of night-time precinct, drinker; country; age range.
3. Outcome variable description: e.g., number of drinks consumed, breath alcohol concentration (BrAC), heavy drinking episode; continuous or categorical.
4. Predictor variable(s) description: e.g., pre-drank, number of friends present, gender; continuous or categorical; event-level (e.g., today) or individual-level (generally); sequence or combination.
5. Type and magnitude of effect estimate(s) and comparison statistic(s): e.g., difference in means or proportions between groups described by combinations of factors, odds ratio, beta-coefficient, interaction coefficient; p-value, 95% confidence interval, probability.
6. Analytical approach used: e.g., multi-level modelling, person-mean centering of event-level predictors, latent class analysis, factor analysis or principal components analysis.
7. Direction of association(s) (for each valid association): e.g., heavier drinking (i.e., risky context) or lighter drinking (i.e., protective context).

The Quality Assessment Tool for Observational Cohort and Cross-Sectional Studies [40] was used by one researcher (OS) to identify potential sources of bias in the design or conduct of the study.

Combinations and sequences that were consistently associated with heavier drinking were compiled into a list summarising ‘risky contexts’ for men, for women and for either gender. Similarly, combinations and sequences associated with lighter drinking were compiled into a list of ‘protective contexts’ for men, women and either. Contexts were divided into categories that denoted whether the context was described by factors relating to the characteristics or state of the individual, physical environments and/or social environments. Specific combinations or sequences that were not unidirectionally associated with the outcome across studies (e.g., a combination was associated with heavier drinking in one study, but lighter drinking in another) were dropped (see S3 Table for dropped associations). A meta-analysis was not conducted because few studies investigated comparable combinations and sequences, thus yielding effect estimates that were not collapsible across a sufficient number of studies.

## 3. Results

### 3.1. Study selection

The flow of records throughout the screening process is presented in Fig 2. The literature database searches retrieved a total of 1,953 records. A further 38 records were retrieved via consultation with experts (‘other sources’). After the removal of 89 duplicate records, the titles and abstracts of 1,902 records were screened according to the eligibility criteria (Table 1). A further 1,661 records were excluded during the screening of titles and abstracts. Of the remaining 241 records which underwent full text screening, 163 were excluded during the full text screening and a further 13 were excluded during critical appraisal or data extraction. The reasons for exclusion during title and abstract screening, full text screening, and critical appraisal and data extraction are listed in Fig 2. The remaining 65 articles were included in the review.

### 3.2. Study descriptions and methodologies

#### 3.2.1. Study sample

Characteristics of the studies included in the review are described in Table 2. The sample size ranged from 47 to 60,215 individuals. However, it is important to note that EMA, daily diary and TLFB [41] designs produce observations for multiple occasions per individual, and EMA designs produce observations for multiple timepoints per occasion per individual. Thus, despite having a low sample size, the data from the EMA, daily diary and TLFB studies included in this review typically encapsulated hundreds of occasions or more. The majority (52/65; 80%) of the studies used data from samples comprised mostly or entirely of adolescents or young adults: students (36/65; 55%); nightlife precinct patrons (12/65; 18%); and people aged < 30 years who were not specifically students or nightlife precinct patrons (5/65; 8%). Twenty-nine studies (45%) restricted their sample to drinkers. Almost all of the studies drew their sample from populations in North America (42/65; 65%) and Europe (17/65; 26%). Participants were primarily of white/Caucasian ethnicity.

**Table 2:** Study characteristics and risk of bias within studies.

Study	Design	Sample		Outcome(s)	Predictor(s)	Effect; comparison	Analysis			Study quality ^a^
		N	Description				MLM	PMC	LCA/FA	
Ally et al., 2016 [42]	RS	60215	Adult drinkers, 18+, Great Britain	6–12 drinks (women), 8–16 (men); >12 (women), >16 (men)	Combination	%; Pr	✓		✓	Good
Arpin et al., 2015 [43]	EMA	47	Adult drinkers, 18+, USA	N drinks (alone at home)	Combination	B, IB; P	✓	✓		Good
Barnett et al., 2013 [44]	DDS	750	Students, <21 years, USA	N drinks; BAC	Sequence, combination	ERR, B, IB; P	✓			Good
Barry et al., 2013 [45]	IS	1029	Nightlife precinct patrons, 18+, USA	BAC	Sequence	B; P				Fair
Bellis et al., 2010 [46]	IS	214	Nightlife precinct patrons, England	N drinks; BAC	Sequence	M; P				Fair
Bersamin et al., 2016 [47]	RS	366	Adolescent drinkers, 15–18, USA	N drinks	Combination	ERR; I; P	✓			Fair
Bourdeau et al., 2017 [48]	IS	615^b^	Nightlife precinct patrons, USA	(Anyone in group): BAC ≥ .05%; BAC ≥ .08%	Combination	M, %; P			✓	Good
Buettner et al., 2011 [49]	RS	3796	Students, 18+, USA	N drinks (bef./at party)	Combination	B, IB; P				Good
Carlini et al., 2014 [50]	IS	1822	Nightlife precinct patrons, 18+, Brazil	BAC ≥ .08% (at exit)	Sequence	OR; P	✓			Good
Cohen et al., 2007 [51]	DDS	193	Adults, 18+, USA	N drinks	Combination	B, IB; P	✓	✓		Good
Clapp et al., 2003 [52]	RS	401	Student drinkers, 18–22, USA	Intoxication level (‘felt drunk’ * N drinks)	Combination	B; P				Fair
Clapp et al., 2008 [53]	IS	1304	Students attending parties, USA	BAC	Combination	B, IB; P	✓			Fair
Dehart et al., 2009 [54]	DDS	505	Students, USA	N drinks	Combination	B, IB; P	✓	✓		Good
Dietze et al., 2017 [55]	RS	710	Risky drinkers, 18–24, Australia	N drinks	Sequence	B; P	✓	✓		Good
Durbeej et al., 2017 [56]	IS	4352	Football match attendees, 16+, Sweden	BAC	Sequence	B; P				Good
Dvorak et al., 2014 [57]	EMA	100	Student risky drinkers, 15–25, USA	Intoxication level (N drinks * intoxication ratings)	Combination	B, IB; P	✓	✓		Good
Fairlie et al., 2015 [58]	DDS	399	Students, <21, USA	11+ drinks (men), 8+ (women); BAC ≥ .16%	Sequence	OR, IOR; P	✓	✓		Good
Finlay et al., 2012 [59]	DDS	717	Students, <21, USA	N drinks; 5+ drinks (men), 4+ (women)	Combination	B, IB, OR, IOR; P	✓			Good
Glindemann et al., 2006 [31]	IS	1337	Nightlife precinct patrons, 18–59, USA	BAC	Sequence, combination	M, I; P				Fair
Groefsema et al., 2016 [60]	EMA	192	Drinkers, 18–25, Netherlands	N drinks	Combination	B, IB; P	✓			Good
Grzywacz et al., 2008 [61]	DDS	802	Adult drinkers, 25+, USA	5+ drinks (men), 4+ (women)	Sequence, combination	B, IB + P	✓			Good
Harford, 1983 [62]	RS	717	Adult drinkers, 18+, USA	N drinks	Combination	M, I; P				Fair
Howard et al., 2015 [63]	DDS	734	Students, <21, USA	5+ drinks	Combination	OR, IOR; P	✓	✓		Good
Hummer et al., 2013 [64]	RS	988	Student risky drinkers, USA	N drinks; BAC	Sequence, combination	M, B, IB; P		✓		Fair
Jackson et al., 2010 [65]	DDS	115	Student smokers and drinkers, 18–19, USA	N drinks	Combination	B, IB; P	✓	✓		Good
Jih et al., 1995 [66]	TLFB	194	Students, USA	N drinks	Combination	M, I; P				Poor
Kairouz et al., 2002 [7]	TLFB	6598	Student drinkers, Canada	N drinks	Combination	B, IB; P	✓			Good
Kuntsche et al., 2013 [35]	EMA	183	Students, Switzerland	5+ drinks (men), 4+ (women)	Sequence, combination	OR, IOR; P	✓			Good
Kuntsche et al., 2015 [67]	EMA	164	Students, Switzerland	Accelerated drinking rate	Sequence, combination	OR; P	✓			Good
Labhart et al., 2013 [33]	EMA	183	Students, Switzerland	N drinks; 5+ drinks (men), 4+ (women)	Sequence	M, %, B; P	✓			Good
Labhart et al., 2014a [68]	EMA	115	Students, Switzerland	N drinks	Sequence, combination	B, IB; P	✓	✓		Good
Labhart et al., 2014b [69]	EMA	183	Students, Switzerland	N drinks off/on premise	Sequence, combination	B, IB; P	✓			Good
LaBrie et al., 2008 [34]	TLFB	238	Student drinkers, USA	N drinks; BAC	Sequence, combination	M, I; P				Fair
Lau-Barraco et al., 2016 [37]	TLFB	238	Non-student risky drinkers, 18–25, USA	N drinks	Combination	B; P	✓			Good
Laws et al., 2017) [70]	DDS	78	Adult drinkers, 18+, USA	N drinks	Combination	B, IB; P	✓	✓		Good
Loxley et al., 1992 [71]	RS	1133	Adult drinkers, 18+, Australia	eBAC (estimated maximum)	Combination	M, I; P				Fair
Luk et al., 2017 [72]	EMA	347	Student drinkers, USA	N drinks	Sequence, combination	B, IB; P	✓	✓		Good
Luoma et al., 2018 [73]	DDS	70	Adult drinkers, 18+, USA	N drinks (alone)	Combination	ERR; I; P	✓	✓		Good
McClatchley et al., 2014 [74]	IS	470	Nightlife precinct patrons, UK	N drinks	Sequence	B, P				Good
Meisel et al., 2017 [75]	RS	972	Student drinkers, <23, USA	N drinks	Sequence	B, P	✓			Fair
Merrill et al., 2013 [76]	TLFB	44	Students, USA	N drinks	Sequence	M; P	✓			Fair
Mohr et al., 2001 [77]	EMA	110	Adult drinkers, 26–44, USA	N drinks (alone; with others; at home; away from home)	Combination	B; P	✓			Good
Mohr et al., 2005 [78]	DDS	122	Student drinkers, USA	N drinks (at home; away from home)	Combination	B; P	✓	✓		Good
Mustonen et al., 2014 [79]	RS	1566	Drinkers, 15–69, Finland	eBAC > .05%; eBAC > .10%	Combination	%; Pr	✓		✓	Good
O'Grady et al., 2011a [80]	DDS	476	Students, USA	N drinks	Combination	B, ERR; P	✓			Fair
O'Grady et al., 2011b [81]	DDS	523	Students, USA	N drinks	Combination	B; P	✓	✓		Fair
O'Hara et al., 2014 [82]	EMA	1636	Students, USA	N drinks	Combination	B, OR; P	✓	✓		Good
Ostergaard et al., 2014 [83]	IS	268	Nightlife precinct patrons, Denmark/UK	N drinks on premise	Sequence, combination	B; P	✓			Fair
Paradis et al., 2011 [84]	RS	403	Male adult drinkers, 18–55, Canada	N drinks	Combination	B, IB; P	✓			Good
Paschall et al., 2007 [85]	TLFB	10152	Students, USA	N drinks (via before + during + after party/ event/ venue)	Sequence, combination	B; P	✓			Good
Patrick et al., 2016 [86]	DDS	72	Students, 18+, USA	N drinks; 5+ drinks (men), 4+ (women)	Combination	OR, IOR, RR, I; P	✓	✓		Fair
Peacock et al., 2016 [87]	IS	5556	Nightlife precinct patron drinkers, 18+, Australia	BAC ≥ .08%	Combination	%; Pr			✓	Good
Pedersen et al., 2007 [88]	TLFB	193	Students, 18–25, USA	N drinks	Sequence, combination	M; CI				Fair
Pennay et al., 2015 [89]	IS	3021	Nightlife precinct patrons, 18+, Australia	BAC	Sequence, combination	B, IB; P	✓	✓		Good
Quigg et al., 2013 [90]	IS	244	Student nightlife precinct patrons, England	N drinks; BAC; BAC ≥ .08%	Sequence	Median, OR; P				Fair
Santos et al., 2015 [91]	IS	1822	Nightlife precinct patrons, 18+, Brazil	BAC (at exit); BAC ≥ .38mg/L (at exit)	Sequence	M, %; P				Fair
Simons et al., 2005 [92]	EMA	56	Student drinkers, 21–23, USA	N drinks	Combination	B, IB; P	✓	✓		Good
Simons et al., 2010 [93]	EMA	102	Student drinkers, 18–24, USA	BAC (at end of occasion)	Combination	B, IB; P	✓	✓		Good
Smit et al., 2015 [94]	EMA	197	Drinkers, 18–25, Netherlands	N drinks	Combination	B, IB; P	✓			Good
Stappenbeck et al., 2015 [95]	EMA	133	Female student drinkers, 18+, USA	N drinks	Combination	B, IB; P	✓	✓		Good
Thrul et al., 2015 [36]	EMA	183	Student drinkers, Switzerland	N drinks at any given time-point of occasion	Combination	B, IB; P	✓			Good
Thrul et al., 2016 [96]	EMA	183	Student drinkers, Switzerland	Drinking rate; Drinking rate acceleration	Combination	B, IB; P	✓			Good
Trim et al., 2011 [38]	DDS	375	Students, USA	5+ drinks (at location)	Combination	B, %; P			✓	Good
Tutenges et al., 2012 [97]	TLFB	110	Tourists, 15–30, Denmark	N drinks	Sequence	B; P	✓			Fair
Wells et al., 2015 [98]	IS	252	Nightlife precinct patrons, 19–29, Canada	N drinks (total); N drinks (venue); BAC (at exit)	Sequence, combination	M, B, IB; P	✓			Good

EMA: ecological momentary assessment (multiple occasions); DDS: daily diary study (multiple occasions); TLFB: timeline follow-back (multiple occasions); IS: Intercept survey (single occasion); RS: Retrospective survey (single occasion); BAC: blood alcohol concentration (estimated breath alcohol concentration or calculated from drinks, time drinking, weight, gender, etc.)

^a^ Judged via the Quality Assessment Tool for Observational Cohort and Cross-Sectional Studies [40]; M: mean; %: percentage/proportion; OR: odds ratio; b: Beta coefficient; IOR: ratio of odds ratios (interaction); IB: interaction coefficient; RR: risk ratio; ERR: event/incident rate ratio; I: Other interaction coefficient; P: p-value; CI: 95% confidence interval; Pr: probability; MLM: multi-level modelling or equivalent to account for the clustering of drinking occasions within individuals; PMC: person-mean centering of event-level predictor(s); LCA: latent class analysis; FA: Factor analysis (or principal components analysis) to derive eligible combination of factors

^b^ 615 groups (1642 individuals).

#### 3.2.2. Study design and analysis

Five different types of study designs were included in the review. Eleven (17%) used a retrospective survey of a single previous drinking occasion, eight (12%) used a TLFB survey design, 14 (22%) used street interviews, 15 (23%) used daily diaries and 17 (26%) used an EMA design. Most studies (47/65; 72%) tested associations via multi-level modelling, accounting for the nested structure of the data, i.e., occasions (level 1) nested within individuals (level 2). Twenty-one studies (32%) used person-mean centered occasion-level predictor variables to aid interpretation of observed effects. Five studies (8%) constructed combinations of factors via latent class analysis or factor analysis.

The study quality (risk of within-studies bias) for most of the studies were rated ‘good’ (44/65; 68%), 20 (31%) were rated ‘fair’, and one (2%) was rated ‘poor’.

#### 3.2.3. Outcomes

Most studies (40/65; 62%) derived an outcome from the number of drinks consumed during the drinking occasion or that day or evening, or from estimated blood alcohol concentration (19/65; 29%). Two studies (3%) combined total drinks consumed during the occasion with ratings of intoxication to derive an outcome measure for intoxication level. Thirteen studies (20%) derived outcome variables from the number of drinks consumed at specific locations/settings (e.g., on premise, off premise, at home, away from home, alone, with others), until specific time-points (e.g., until nightclub entry) or during specific time-frames (e.g., 8pm-9pm, 9pm-10pm, etc.), or speed of consumption (e.g., drinking rate or drinking rate acceleration).

### 3.3. Synthesis of results: Contexts that encourage and discourage heavy drinking

The contexts that were found to be associated with heavier drinking are summarised in Table 3 (denoted via ↑). Contexts associated with lighter drinking are denoted via ↓.

**Table 3:** Contexts–described by combinations and sequences of factors related to the characteristics or state of the individual, the physical environment and the social environment ^1^ –associated with heavier drinking (↑) and lighter drinking (↓) ^2^.

Combinations and sequences of factors (i.e., the context)				All	Men	Women
*Individual characteristics/state*						
	Negative mood					
		(Yesterday) [72]		↑		
		(Accumulation of stress over last 3 days) [61]		↑		↑
		(Accumulation of stress over last 3 days) + < high school education [61]		↑		
		(Stress today) + < high school education [61]		↑		
		(Anxiety today) + high negative urgency/low positive urgency generally [93]		↑		
		(Anxiety today) + high drinking to cope motives generally [82]		↑		
		(Anger today) + low drinking to cope motives generally [82]		↑		
		(Loneliness today) [43]				↑
		(Loneliness today) + high social support generally [43]		↑		
		(Shame today) + high shame generally [73]		↑		
		(Today) + non-student [86]		↓		
		(Stress today) + student unaffiliated with fraternity/sorority [72]		↓		
		(Stress yesterday) + student unaffiliated with fraternity/sorority [72]		↓		
		(Shame today) + low shame generally [73]		↓		
		(Anxiety today) + low sustained attention generally [57]			↓	
		(Anxiety today) + high sustained attention generally [57]				↓
	Positive mood today					
		+ Non-student [86]		↑		
	Reason for drinking today					
		To be social, to comply with others, to feel good, to relax, get drunk, or to celebrate [7]			↑	
	Alcohol consumption yesterday					
		(Lighter than usual) [68]		↑		↑
		(Lighter than usual) + low alcohol consumption generally [68]		↑		
		(Heavier than usual) [68]		↓		↓
		(Heavier than usual) + low alcohol consumption generally [68]		↓		
	Cigarette consumption today [65]					↑
		(Cigarettes) + light smoker generally [65]		↑		
		(Any cigarettes) + heavy but non-daily smoker generally [65]		↑		
*Physical environment*						
	Locations/activities today					
		Number of drinking locations [55]		↑		
		Number of party-related tour activities [97]		↑		
*Social environment*						
	Pre-drinking today					
		Pre-drink/pre-game/pre-party [33–35, 44–46, 50, 56, 58, 64, 67, 69, 74–76, 83, 88–91, 98]		↑		
		With group who had pre-drank [98]		↑		
		Pre-drink today + with a group who had pre-drank today [98]		↑		
	Intentions today					
		Intend to get drunk + with friends you believe intend to get drunk today [52]		↑		
	Social rejection today + with close others today [70]			↑		
	Driver today					
		Driver to drinking setting today + not driver returning from drinking setting today [71]		↑		
		Driver to drinking setting today + driver returning from drinking setting today [71]		↓		
	Drinking group today					
		Low expectation for drinking + low expectation for illicit drug use + no illicit drug use + no impaired drivers + no experiences of sexual aggression + older + not a special occasion + romantic couple among group + low closeness of group members + small drinking group [48]		↓		
	Weekday/weekend today					
		(Weekday) + spiritual activities today [59]		↓		
		(Weekend) + athletics activities today [59]		↓		
*Individual characteristics/state x physical environment*						
	Attend event today					
		(Themed party) [53]				↑
		(Fraternity/sorority party) [85]			↑	
		(Campus event) [85]			↑	
		(Off-campus party) [85]			↑	
		(Get-together) + am a parent [84]			↑	
	Energy drink consumption today					
		+ short drinking session today [89]		↑		
	Main location of drinking today					
		(Own or other’s home) + increased access to alcohol [47]			↑	
		(Own home) [47]				↓
		(Home this evening) + positive mood today + high drinking to cope motives generally [78]		↑		
		(Home this evening) + positive mood today + high drinking to enhance motives generally [78]		↑		
		(Home this evening) + negative mood today + high drinking to cope motives generally [78]		↑		
		(Home this evening) + negative mood today + low drinking to cope motives generally [78]		↑		
		(Restaurant) [7]			↓	
*Individual characteristics/state x social environment*						
	Pre-drink today					
		(Yes) + Sophomore/older (vs. freshman/younger)] [44]		↑		
		(Yes) + drank straight spirits today [69]				↑
		(Yes) + drank wine/champagne today [69]			↑	
		(Yes) + illicit drug use today [87]			↑	
		(Yes) + mixed gender setting today [64]			↑	
		(Yes) + only same-sex friends present today [69]			↑	
		(Yes) + lower conformity motives generally [35]			↑	
		(No) + drinking games today [64]		↑	↑	
	Social group today					
		Many friends present [36]			↑	
			(Either sex friends) + high coping motives generally [96]			↑
			(Opposite-sex friends) + high social (vs. non-social) alcohol attentional bias generally [60]	↑		
			(Opposite-sex friends) + high coping motives generally [94]		↑	
			(Opposite-sex friends) + low conformity motives generally [94]		↑	
			(Opposite-sex friends) + high enhancement motives generally [94]			↑
			(Same-sex friends) + low coping motives generally [94]		↑	
			(Same-sex friends) + high social (vs. non-social) alcohol approach bias generally [60]			↑
		Less friends present [7]			↓	
			(Same-sex friends) + strong non-social (vs. social) alcohol attentional bias generally [60]			↓
		With others who are drinking a lot [81]			↑	
		Drinking group today				
			High expectation for drinking + illicit drug use + high assessment of safety [48]	↑		
			High expectation for drinking + illicit drug use + impaired driver among group [48]	↑		
			High expectation for drinking + high discrepancy for expectation of drinking + illicit drug use + high discrepancy for assessment of safety + experienced physical aggression + large drinking group [48]	↑		
			Low expectation for drinking + no illicit drug use + low assessment of safety + no impaired drivers + no experiences of sexual aggression + many part-time or un-employed + all straight females [48]			↓
	Weekday/weekend today					
		(Weekend) [59]				↑
		(Weekend) + high social expectancies generally [37]		↑		
		(Weekend) + positive mood today + older (later in college career) [63]		↑		
		(Weekday) + positive mood generally + older (later in college career) [63]		↑		
		(Weekday) [37]				↓
		(Weekday) + younger [37]		↓		
		(Weekday) + low harmfulness of drinking generally [37]		↓		
		(Weekday) + low social alcohol expectancies generally [37]		↓		
		(Weekday) + positive mood generally + younger (earlier in college career) [63]		↓		
		(Weekend) + negative mood today + started drinking at very young age [63]		↓		
	Positive/negative/neutral interpersonal events/situations/exchanges today					
		(Negative) + alone this evening + high neuroticism generally [77]		↑		
		(Negative) + alone this evening + high extraversion generally [77]		↑		
		(Negative) + alone this evening + low neuroticism generally [77]		↑		
		(Negative) + low implicit self-esteem generally [54]		↑		
		(Negative–embarrassing) + socially anxious generally [80]		↑		
		(Negative–high sexual assault distress) + low distress coping control generally [95]				↑
		(Positive) + alone this evening + high neuroticism generally [77]			↑	
		(Positive) + alone this evening + low neuroticism generally [77]		↓	↓	
		(Positive) + older (university student vs. college student) [66]		↑		
		(Positive) + high implicit self-esteem generally [54]		↑		
		(Positive–low sexual assault distress) + high distress coping control generally [95]				↑
		(Neutral) + low social integration generally [51]		↑		
		(Negative–embarrassing) + Not socially anxious person generally [80]		↓		
	Not driver returning from drinking setting today [71]				↑	
	Consumption today					
		(Illicit drugs) + long duration of drinking session today [89]		↓		
*Physical environment x social environment*						
	Location/event today					
		Off-campus party today + party host today [49]		↑		
		On campus party today + party attendee (not host) today [49]		↑		
		Drink in bar today + pre-drink today [31]		↑		
		Drink at own home today + many people present [47]		↑		
		Home location + 6pm-12am (midnight) + weekend + friends [79]		↑		
		Licensed venue location + 6pm-12am [79]		↑		
		Licensed venue location + weekend + with friends + > 4 people in group + meeting friends [79]		↑		
		Public location + many people intoxicated [38]		↑		
		Private location + many people intoxicated [38]		↑		
		At home this evening + high time with friends today + positive interpersonal exchanges today [78]		↑		
		At home this evening + low time with friends today + negative interpersonal exchanges today [78]		↓		
		Public location + few people intoxicated [38]		↓		
		Private location + few people intoxicated [38]		↓		
	Drinking occasion today					
		Own home location + < 1-hour duration + with family + with mixed-sex group [42]		↓		
		Home location + 6pm-12am (midnight) + alone + no special occasion [79]		↓		
	Illegal drugs available + many people intoxicated today + played drinking games [52]			↑		
*Individual characteristics/state x physical environment x social environment*						
	Location/event today					
		Drink in bar today + pre-drink today + younger [31]		↑		
		Drink in setting other than home/bar/restaurant today + with spouse/relatives today [62]				↑
		Drink in bar today + with friends today [62]				↑
		Public location + many people intoxicated + not in committed relationship [38]		↑		
		Private location + many people intoxicated + not in committed relationship + intention to get drunk [38]		↑		
		Drink at home today + with spouse/relatives today [62]				↓
		Drink at own/other’s home today + responsible adult present [47]				↓
		Drink at own/other’s home today + many males present [47]			↓	
		Private location + few people intoxicated + in committed relationship + no intention to get drunk [38]		↓		
		Public location + few people intoxicated + in committed relationship [38]		↓		
		At home this evening + negative interpersonal exchanges today + high neuroticism generally [77]		↑		
		At home this evening + negative interpersonal exchanges today + high extraversion generally [77]		↑		
		At home this evening + negative interpersonal exchanges today + high drinking to cope motives generally [78]		↑		
		At home this evening + negative interpersonal exchanges today + low drinking to cope motives generally [78]		↓		
		At home this evening + negative interpersonal exchanges today + low drinking to conform motives generally [78]		↑		
		At home this evening + positive interpersonal exchanges today [78]			↑	↓
		At home this evening + positive interpersonal exchanges today + high drinking to cope motives generally [77]				↑
		At home this evening + positive interpersonal exchanges today + low drinking to cope motives generally [77, 78]		↑	↑	
		At home this evening + positive interpersonal exchanges today + low drinking to enhance motives generally [78]		↑		
		At home this evening + positive interpersonal exchanges today + high drinking to enhance motives generally [78]		↓		
		At home this evening + fewer positive interpersonal exchanges today + high drinking to cope motives generally [77]			↑	
		At home this evening + high time spent with friends today + low drinking to cope motives generally [78]		↑		
		At home this evening + high time spent with friends today + low social drinking motives generally [78]		↑		
		At home this evening + high time spent with friends today + low drinking to enhance motives generally [78]		↑		
		Away from home this evening + positive interpersonal exchanges today + high extraversion generally [77]			↑	↓
		Away from home this evening + positive interpersonal exchanges today + low extraversion generally [77]		↓		
		Away from home this evening + positive interpersonal exchanges today + low neuroticism generally [77]		↓		
		Away from home this evening + positive interpersonal exchanges today + low social drinking motives generally [78]		↑		
		Away from home this evening + negative interpersonal exchanges today + low drinking to conform motives generally [78]		↑		
		Away from home this evening + high time spent with friends today + low drinking to enhance motives generally [78]		↑		
	Drinking occasion today					
		Drink beer + own home location + start 5pm-8pm + 1–3 hours duration + weekend + with friends + with mixed-sex group [42]		↑		
		Drink off-premise wine + own home location + start 5pm-8pm + 1–3 hours duration + with spouse/partner + with mixed-sex group [42]		↑		
		Drink off-premise wine + own home location + < 1-hour duration + with spouse/partner + with mixed-sex group [42]		↓		
		Meal + home location + 7am-6pm + with spouse/partner [79]		↓		

Heavier drinking: Higher event-level alcohol consumption; Lighter drinking: Lower event-level alcohol consumption

^1^ Must include at least one context-level variable and must not include event-level alcohol consumption (the outcome)

^2^ Compared to the reference categories of categorical variables and/or the lower values of continuous variables (unless inverted or transformed), as derived by the authors; ‘Today/yesterday’: event-level variable; ‘Generally’: Individual-level variable; Single factor + arrow in ‘all’ column: sequence; All: among sample of men and women combined; Single factor + arrow in gender column: effect of that single factor greater for that gender than the other gender (i.e., gender interaction) (does not refer to effect of one factor among sample of women or men); Combinations/sequences allocated to the most relevant subsection according to the types of factors involved (subsections in *italics*).

#### 3.3.1. Coverage of contextual elements

A total of 156 unique contexts were identified as being associated with heavier or lighter drinking. Of these, 110 contexts (71%) were associated with heavier drinking (labelled as ‘risky contexts’), and 46 contexts (29%) were associated with lighter drinking (labelled as ‘protective contexts’).

Twenty-eight studies (43%) investigated the association between a sequence of event-level factors and event-level drinking, and 52 studies (80%) investigated the association between a combination of two or more context-related factors and event-level drinking. The number of studies that constructed sequences or combinations from factors from the following domains were as follows:

- individual characteristics/state only, 11/65 (17);
- physical environment only, 2/65 (3%);
- social environment only, 25/60 (38%);
- individual characteristics/state and physical environment, 7/65 (11%);
- individual characteristics/state and social environment, 21/65 (32%);
- physical environment and social environment, 8/65 (12%);
- individual characteristics/state and physical environment and social environment, 8/65 (12%).

Most contexts were described from combinations or sequences of factors related to the individual (128/156; 82%) or social environment (113/156; 72%), exclusively or in combination with individual, social environment or physical environment factors. Fewer contexts were described from factors related to the physical environment, exclusively or in combination with individual or social environment factors (68/156; 44%). Twenty-four risky contexts and thirteen protective contexts (total 37/156; 24%) were described from a combination of factors related to all three elements of a context: the individual, the social environment and the physical environment.

#### 3.3.2. Individual’s state (e.g., daily mood)

Negative or positive states of mood were found to be associated with heavier or lighter drinking depending on the individual’s traits and environmental characteristics that these emotions are combined with [57, 61, 63, 72, 82, 86, 93]. Heavy drinking is particularly likely to occur on days when a lot of negative emotion or negative interpersonal events are experienced for individuals who are socially anxious [80], have low self-esteem, high shame or high neuroticism [54, 73, 77], have high social support [43], are less educated [61], have high drinking-to-cope motives [78, 82] or have low drinking to conform motives [78]. In contrast, studies suggested that people with low shame [73], non-students [86], students not affiliated with a sorority [72], men with low attention spans [57] and women with high attention spans [57] are less likely to engage in heavy drinking on days they have negative mood. Also, individuals with low social anxiety or low drinking to cope motives tend to drink less on days they experience negative interpersonal interactions [78, 80]. Individuals with relatively high self-esteem [54], high perceived ability to cope with distress [95] or who are older [66] are particularly likely to engage in heavy drinking on days when they are in a positive mood or experience positive interpersonal events.

#### 3.3.3. Social characteristics of contexts (e.g., the drinking group)

The immediate social context was found to be most strongly associated with drinking behaviour when combined with certain individual characteristics. Heavy drinking occasions are more likely on occasions when many friends are present [36, 42, 47, 48, 52, 60, 62, 70, 78, 94, 96]–particularly for those with high attention to social aspects of drinking (i.e., cognitive bias towards social vs. non-social drinking situations) [60], when drinking at home [47, 78], for men with high coping drinking motives or low conformity motives when many female friends are present [94], for men with low coping motives when many male friends are present [94], for women with high enhancement motives when many male friends are present [94], and for women who have a cognitive bias for social alcohol-related stimuli when many female friends are present [60]. Drinking with a group of friends was associated with very heavy drinking when the group’s expectations of drinking and illicit drug use are high [48]. Logically, when the group expectations of drinking and illicit drug use are lower, this was associated with lighter drinking [48]. Having less friends present was identified as a particularly strong protective context for men in general [7], and for women who have a cognitive bias for non-social alcohol-related stimuli [60].

Certain contexts appear particularly conducive to heavy drinking on weekends. Generally, spending time with friends, particularly in large groups of friends, is associated with heavier drinking [42, 79]. On average, women, individuals with social expectancies related to alcohol, and older college students (fourth year vs. first year college students) who are generally in a positive mood are likely to drink much heavier on weekends than weekdays. On a day-to-day basis, older college students are particularly likely to drink heavily on weekend days if they are in a positive mood that day [63].

Some contexts experienced at a given time of the week are associated with lighter than usual drinking. Students who initiated drinking at an early age are less likely to engage in heavy drinking on weekend days if they are in a negative mood [63]. Also, engaging in spiritual activities on weekdays and engaging in athletics activities such as sports on weekends were contexts associated with lighter drinking than usual on weekdays and weekends, respectively [59].

#### 3.3.4. Physical characteristics of contexts (e.g., location)

In particular circumstances, both public locations (e.g., bars) and private places (e.g., homes) may be conducive to heavy drinking. Occasions spent with groups of friends or in environments where many people are intoxicated were associated with heavy drinking regardless of whether they were in a public or private location [38]. Generally, social events such as parties are relatively likely to involve heavy drinking [49, 53, 84, 85, 97]. The increased likelihood of engaging in heavy drinking when attending a party is particularly large when the individual is hosting an off-campus party [49], for men when attending a University-related event or party [85] and for women when attending a themed party [53]. In contrast, when men attend a restaurant they are relatively unlikely to drink heavily [7]. Heavy drinking was more likely when spending the evening at home in a negative mood or on days involving negative interpersonal exchanges, especially among highly neurotic or extraverted people [77]. Heavy drinking was more likely for those with high drinking to cope and drinking to enhance motives when spending the evening at home in a positive mood, or on days when spending a lot of time with friends [78].

#### 3.3.5. Sequences of context-related factors

There were three main sequences that are each found to be associated with heavy drinking: pre-drinking, yesterday’s alcohol consumption and multi-day accumulation of stress. Multiple studies found that heavy drinking was more likely on occasions involving pre-drinking than non-pre-drinking occasions [31, 33–35, 44–46, 50, 56, 58, 64, 67, 69, 74–76, 83, 87–91, 98]. The positive association between pre-drinking and heavy drinking was particularly strong for women when combined with drinking spirits [69], for men when with a mixed gender or all same-sex group (vs. all opposite-sex) [64, 69], for men when illicit drugs are consumed [87], for men when drinking wine or champagne [69], and for men with low drinking to conform motives [35]. Occasions when an individual pre-drinks were particularly conducive to heavy drinking when the on-premise venue attended is a bar, especially for younger adults [31]. This effect was stronger among women than men and among generally lighter than heavier drinkers. Heavy drinking was also more likely after sequences of stressful days, particularly among women in general or among men or women with a lower level of education [61]. Overall, aside from pre-drinking, very few sequences of factors were reported as being associated with heavier or lighter drinking.

## 4. Discussion

### 4.1. Associations between context and heavy drinking, policy implications, and opportunities for research

Elements of contexts that were commonly associated with heavier or lighter drinking occasions included a person’s mood throughout the day, the size, gender and expectations of the social group, the location where drinking takes place, and whether certain events or parties are attended. However, the strength and direction of associations between context-level factors and drinking differ according to the characteristics of the individual (e.g., their gender, age, personality and motives). The variety of interactions among the elements of a context demonstrate the complexity of relationships between contexts and drinking behaviours. In fact, in a given social or physical context, some people may feel compelled to continue or accelerate their drinking, whereas others may be influenced to slow down, drink less, or choose not to drink at all. Therefore, it is important to consider the physical, social and individual elements of a context when endeavouring to understand contextual influences on people’s drinking. This is also an important consideration for interventions designed at the individual-level. Identification of contexts that are risky or protective for people of a particular gender and age and with particular personality traits and drinking motives may help to design and implement effective policies for reducing heavy drinking occasions and related harms among specific sub-populations.

Identifying contexts that are associated with heavy drinking and contexts associated with lighter drinking is useful because it enables targeted interventions and policies, thus potentially reducing heavy drinking and alcohol-related harms. Concurrent discouragement of risky contexts (via policies, targeted interventions, health promotion and education) and encouragement of protective contexts (via the same avenues) may further reduce heavy drinking and alcohol-related harms.

The search strategy retrieved 31 studies that are well-suited to measuring sequences of factors across an occasion (17 EMA studies and 14 intercept surveys). However, very few studies investigated links between specific sequences during an occasion and event-level drinking, aside from pre-drinking. It is also possible that some eligible studies that investigate sequences of factors during an occasion and event-level drinking were not captured by the literature search. One of the advantages of EMA studies is they can record the time of specific occurrences across the course of a drinking occasion [99]. Therefore, new EMA studies or analysis of existing EMA data are needed to identify specific sequences that influence young adults to accelerate, maintain, decelerate, or cease drinking on a given occasion.

Relatively few contexts were described in relation to the physical environment. Research investigating whether and how physical contexts are associated with an individual’s drinking behaviour is needed because, in a practical sense, modification of the physical environment may be relatively feasible (e.g. luminosity, noise level and density restrictions, location-specific text message interventions) [100]. Technologies such as smartphone-based environmental measurement tools may be useful for measuring physical environments and investigating associations with heavy drinking behaviours [101–103].

A minority of the studies included in this review described contexts via a combination of factors related to the individual, the social environment and the physical environment. Future event-level studies that consider all the main elements that comprise a context have the potential to improve understandings of how and why specific contexts can influence drinking behaviour.

### 4.2. Study design and analytical methodology: implications and opportunities for studying drinking contexts

Numerous types of event-level or event-based designs were used by the studies included in this review. However, their ability to comprehensively describe contexts and explore their associations with event-level drinking is varied.

To accurately distinguish between the contextual influences at the event-level and the individual-level, studies which capture information about multiple occasions, such as EMA and daily diary studies are advantageous because they can properly account for both inter- and intra-individual variations using multi-level modelling [104, 105]. Studies that account for inter- and intra-individual variations using multi-level modelling can explore the true effects of contexts as drivers of within-person variation in event-level drinking [104, 105]. Multi-level modelling also enables event-level predictor variables to be centered according to each individual’s mean measurement across all occasions (called person-mean centering). Person-mean centering uses the person’s usual behaviour as its own baseline, and standardizes the momentary behaviour by considering only the deviation from the usual behaviour [105].

Studies with longer recall periods, such as retrospective surveys about single occasions and TLFB surveys, are more susceptible to recall bias [106].

Event-level studies may harness new technologies such as smartphone environmental measurement tools and continuous objective monitoring of alcohol use to improve measurement of contextual factors and behaviours. Continuous objective monitoring of blood alcohol concentration in real time via objective measures such as transdermal sensors may reduce the risk of self-reporting biasing measurements of alcohol consumption and intoxication levels [107]. Smartphones may provide useful tools for objectively measuring other contextual factors and behaviours [100, 101, 103, 108]. Although, data gathered via built-in sensors, camera, microphone and other features are not without limitation and subjective self-report questionnaires may temporarily remain the most practical method for measuring many contextual factors and behaviours.

This review found that most studies that explore event-level associations between contexts and drinking were conducted among samples of adolescents and young adults, and often students. Therefore, many of the risky and protective contexts identified may generalise to younger populations, but not to older populations. The scope of this review was widened from young adults to all ages during the project conception and design phase, to attain a more-complete review of the relevant literature and to compare the representation of young samples and older samples in the literature on drinking contexts. The high representation of young samples in the literature appears warranted, given that heavy drinking occasions are most frequent and intense during late adolescence and early adulthood [3, 109, 110]. Although, a proportion of older adults are heavy drinkers and, recently in many countries, alcohol consumption levels have decreased in younger age groups but have been maintained or increased in some older age groups [1, 111–114]. Thus, further exploration of event-level associations between contexts and drinking among older populations may be warranted to identify and discourage contexts associated with heavy drinking in middle-late adulthood. Further, as all studies included in the review sampled from Western countries, the results may generalisable to younger populations in Western countries only.

Similarly, the results of this review may only generalise to broad Western populations, not to socio-demographically-defined sub-populations, which are known to vary in alcohol use and problems. Therefore, research investigating event-level associations between contexts and heavy drinking in specific sub-populations of both Western and non-Western countries is necessary.

The intuitive way to construct variables and interpret model estimates may partly explain why fewer protective contexts were identified than risky contexts. The reference category of a categorical variable or the lower value of a continuous variable typically corresponds with a lower level of exposure–e.g., a categorical variable for pre-drinking with reference category ‘no’ and comparison category ‘yes’. Also, the format of effect estimates of regression models (e.g., odds ratios, beta coefficients) make it intuitive to interpret associations as the comparison category (or higher value) versus the reference category (or lower value)–e.g., ‘pre-drinking is associated with heavy drinking’. The same effect estimate could also be interpreted as the reference category versus the comparison category–e.g., ‘not pre-drinking is associated with lighter drinking’. Both interpretations are correct and provide a slightly different interpretation, although the former (e.g., comparison category vs. reference category) is more intuitive and commonly reported. While in some cases it is possible to derive protective contexts by taking the reverse of the reported association (e.g., ‘did not pre-drink’), we chose not to do so because this is generally not how estimates are interpreted and explained in literature. Thus, the tendency for lower values to be a variable’s reference category/value may partly explain why fewer protective contexts were identified than risky contexts.

This review identified many well-designed studies that investigated event-level associations between contexts and drinking using appropriate analytical methodology. However, some studies were highly susceptible to recall bias, and the absence of multi-level modelling in 18 studies meant some studies may not have properly accounted for between-person differences that may bias event-level effects [104, 105].

### 4.3. Strengths and limitations

Traditionally, most event-level and event-based studies of drinking contexts have focussed on describing independent associations between singular isolated context-related factors and drinking outcomes. This review excludes a section of the literature that does not conceptualise and measure contexts as combinations or sequences of factors. As drinking contexts are described by the complex interaction of factors related to the physical and social environment and the individuals within them [5, 6, 8–10], these associations were not eligible for inclusion in this review. Therefore, there are likely numerous studies not included in this review that take a simpler approach and identify context-related factors which are independently associated with heavy drinking. However, the focus of this review on more complex conceptualisation and measurement of drinking contexts provides a novel and useful review of the cutting-edge of event-level alcohol research. Given the complex conceptualisation of drinking context employed by this review, developing a search strategy that effectively captures the relevant literature was difficult and a potential limitation. The terms used to identify event-level or event-based study design did not list specific study designs (e.g., timeline follow back, ecological momentary assessment) (S2 Table). An objective of this review was to summarise and critique the study design of the event-level drinking literature, and inclusion of such terms would bias the search to capturing study designs that are well known to the researchers a-priori. Experimental studies were not included in this review because they do not represent real-world settings but provide another method for studying drinking contexts. Screening and data extraction were conducted by an independent researcher. To minimise this limitation, records that the researcher was initially unsure whether to include or exclude were screened for eligibility by a second researcher, and differences in opinions of eligibility were resolved via the majority opinion of a group of five researchers. While event-level alcohol consumption and intoxication (the review’s outcome measure) is associated with alcohol-related harms, this review does not explore direct associations with between contexts and alcohol-related harms. While qualitative literature was not included in this review, such studies provide clues as to the types of contexts that may be associated with heavy drinking and related harms. Therefore, there may be some context-related factors associated with occasions involving heavy drinking or harms not captured in this review that warrant further exploration.

### 4.4. Conclusions

This review found that the contexts an individual encounters on a given occasion are associated with how heavily they drink alcohol during that occasion. The direction and magnitude of these associations differ according to the gender, age, personality, motives and mental state of the individual, such that in a given social or physical context some people may feel compelled to continue or accelerate their drinking whereas others may be influenced to slow down, drink less, or choose not to drink at all. Contexts or factors are experienced in specific sequences that shape the broader drinking context and influence drinking behaviours and alcohol-related consequences across drinking occasions. However, risky contextual sequences are under-studied. Therefore, event-level alcohol research should prioritise improving understandings of the types and mechanisms of contextual sequences that are associated with heavy drinking and alcohol-related harms. New technologies such as smartphone environmental measurement tools and continuous objective monitoring of alcohol use and multi-level analytical methods are recommended to improve understandings of why people engage in heavy drinking. Continued research investigating event-level associations between contexts and heavy drinking will facilitate public health interventions and policies that reduce heavy drinking and alcohol-related harms.

## Supporting information

S1 Table PRISMA checklist. ^a^ Of submitted version of manuscript document; n.p.: Not possible (see page 10: ‘a meta-analysis was not conducted because few studies investigated comparable combinations and sequences, thus yielding effect estimates that were not collapsible across a sufficient number of studies’); S1 Table adapted from: [1].
(DOCX)

S2 Table Search strategy used to retrieve articles from scientific literature databases to be screened for eligibility. Databases searched: MEDLINE, Embase and the Cumulative Index to Nursing and Allied Health Literature (CINAHL); Searched titles and abstracts of records; The above search terms were applied in MEDLINE, and replicated in Embase and CINAHL (via some minor adjustments); Search limits: Exclude MEDLINE records (applied to Embase and CINAHL searches only); Executed search strategy and retrieved articles on 29 January 2018.
(DOCX)

S3 Table Contexts–described by combinations and sequences of factors related to the characteristics or state of the individual, the physical environment and the social environment ^1^ –associated with heavier drinking (↑) or lighter drinking (↓) ^2^ that were dropped due to inconsistent observed direction of association across studies. Heavier drinking: Higher event-level alcohol consumption; Lighter drinking: Lower event-level alcohol consumption; ^1^ Must include at least one context-level variable and must not include event-level alcohol consumption (the outcome); ^2^ Compared to the reference categories of categorical variables and/or the lower values of continuous variables (unless inverted or transformed), as derived by the authors; ‘Today/yesterday’: event-level variable; ‘Generally’: Individual-level variable; Single factor + arrow in ‘all’ column: sequence; All: among sample of men and women combined; Single factor + arrow in gender column: effect of that single factor greater for that gender than the other gender (i.e., gender interaction) (does not refer to effect of one factor among sample of women or men); Combinations/sequences allocated to the most relevant subsection according to the types of factors involved (subsections in *italics*).
(DOCX)

S1 File PROSPERO International prospective register of systematic reviews review protocol. (PDF)
