# Supplementary material for: The contexts of heavy drinking: A systematic review of the combinations of context-related factors associated with heavy drinking occasions
Source: PLoS One. 2019 Jul 10;14(7):e0218465. doi: 10.1371/journal.pone.0218465 (PMC6619678; doi:10.1371/journal.pone.0218465)
Supplement: S2 Table — Databases searched: MEDLINE, Embase and the Cumulative Index to Nursing and Allied Health Literature (CINAHL); Searched titles and abstracts of records; The above search terms were applied in MEDLINE, and replicated in Embase and CINAHL (via some minor adjustments); Search limits: Exclude MEDLINE records (applied to Embase and CINAHL searches only); Executed search strategy and retrieved articles on 29 January 2018. (DOCX) [file pone.0218465.s002.docx]

**S2 Table. Search strategy used to retrieve articles from scientific literature databases to be screened for eligibility.**

| **Concept** | **Search #** | **Search terms** |
| --- | --- | --- |
| Alcohol drinking | 1 | (drink* adj2 (risk* or excess* or heavy or heavily or hazard* or binge or harm* or problem* or consequenc*)).tw. |
|  | 2 | (alcohol adj3 (drink* or use* or intake or risk* or consum* or intoxicat* or excess* or hazard* or harm* or problem* or consequenc*)).tw. |
|  | 3 | (risky single occasion drinking or rsod).tw. |
|  | 4 | exp alcoholic intoxication/ or exp binge drinking/ or exp alcohol drinking/ |
|  | 5 | 1 or 2 or 3 or 4 |
| Event-level or event-based study design | 6 | (evening* or weekend* or night* or friday* or saturday* or event or events or party).tw. |
|  | 7 | (drink* adj1 (session* or occasion*)).tw. |
|  | 8 | 6 or 7 |
| Combinations, interactions or sequences (of factors) | 9 | (combination* or co-occur* or conjunction* or interact* or moderates or moderated or moderator* or moderation or modifier* or modifies or modified or modification).tw. |
|  | 10 | ((differ* or stronger or weaker) adj3 (relationship* or associat* or effect)).tw. |
|  | 11 | sequence*.tw. |
|  | 12 | ((before or after) adj3 (venue or location or context or setting or bar or home or club or pub or party)).tw. |
|  | 13 | (predrink* or preload* or pregame or pregaming or preparty* or afterdrink* or afterload* or backload* or sideload*).tw. |
|  | 14 | ((pre or after or back or side) adj1 (drink* or load* or game or gaming or party*)).tw. |
|  | 15 | ((pub or bar) adj1 (crawl* or hop or hopp*)).tw. |
|  | 16 | 9 or 10 or 11 or 12 or 13 or 14 or 15 |
|  | 17 | 5 and 8 and 16 |

Databases searched: MEDLINE, Embase and the Cumulative Index to Nursing and Allied Health Literature (CINAHL); Searched titles and abstracts of records; The above search terms were applied in MEDLINE, and replicated in Embase and CINAHL (via some minor adjustments); Search limits: Exclude MEDLINE records (applied to Embase and CINAHL searches only); Executed search strategy and retrieved articles on 29 January 2018.
