# Supplementary material for: The contexts of heavy drinking: A systematic review of the combinations of context-related factors associated with heavy drinking occasions
Source: PLoS One. 2019 Jul 10;14(7):e0218465. doi: 10.1371/journal.pone.0218465 (PMC6619678; doi:10.1371/journal.pone.0218465)
Supplement: S3 Table — Heavier drinking: Higher event-level alcohol consumption; Lighter drinking: Lower event-level alcohol consumption; 1 Must include at least one context-level variable and must not include event-level alcohol consumption (the outcome); 2 Compared to the reference categories of categorical variables and/or the lower values of continuous variables (unless inverted or transformed), as derived by the authors; ‘Today/yesterday’: event-level variable; ‘Generally’: Individual-level variable; Single factor + arrow in ‘all’ column: sequence; All: among sample of men and women combined; Single factor + arrow in gender column: effect of that single factor greater for that gender than the other gender (i.e., gender interaction) (does not refer to effect of one factor among sample of women or men); Combinations/sequences allocated to the most relevant subsection according to the types of factors involved (subsections in italics). (DOCX) [file pone.0218465.s003.docx]

**S3 Table. Contexts – described by combinations and sequences of factors related to the characteristics or state of the individual, the physical environment and the social environment ^1^ – associated with heavier drinking (↑) or lighter drinking (↓) ^2^ that were dropped due to inconsistent observed direction of association across studies.**

| Combinations and sequences of factors (i.e. the context) | | | All | Men | Women |
| --- | --- | --- | --- | --- | --- |
| *Individual characteristics/state* | | |  |  |  |
|  | Positive mood today | |  |  |  |
|  |  | + High distress tolerance generally [1] | ↑ |  |  |
|  |  | + Low distress tolerance generally [1] | ↑ |  |  |
|  | | |  |  |  |
| *Individual characteristics/state x social environment* | | |  |  |  |
|  | Pre-drink today | |  |  |  |
|  |  | + Man [2-4] |  | ↑ |  |
|  |  | + Woman [5] |  |  | ↑ |
|  |  | + Younger [4] | ↓ |  |  |
|  | Weekday/weekend today | |  |  |  |
|  |  | (Weekday) + negative mood generally + started drinking young age  [6] | ↑ |  |  |
|  |  | (Weekday) + negative mood generally + started drinking at very late age  [6] | ↓ |  |  |
|  | | |  |  |  |
| *Physical environment x social environment* | | |  |  |  |
|  | (Drinking occasion today) | |  |  |  |
|  |  | Own home location + 6pm-12am + weekend + with spouse/partner  [7] | ↑/↓ |  |  |

Heavier drinking: Higher event-level alcohol consumption; Lighter drinking: Lower event-level alcohol consumption; ^1^ Must include at least one context-level variable and must not include event-level alcohol consumption (the outcome); ^2^ Compared to the reference categories of categorical variables and/or the lower values of continuous variables (unless inverted or transformed), as derived by the authors; ‘Today/yesterday’: event-level variable; ‘Generally’: Individual-level variable; Single factor + arrow in ‘all’ column: sequence; All: among sample of men and women combined; Single factor + arrow in gender column: effect of that single factor greater for that gender than the other gender (i.e., gender interaction) (does not refer to effect of one factor among sample of women or men); Combinations/sequences allocated to the most relevant subsection according to the types of factors involved (subsections in *italics*).

**References**

1. Simons J, Gaher R, Oliver M, Bush J, Palmer M. An experience sampling study of associations between affect and alcohol use and problems among college students. Journal of Studies on Alcohol. 2005;66(4):459-69. PubMed PMID: 16240553.

2. Barnett N, Orchowski L, Read J, Kahler C. Predictors and consequences of pregaming using day- and week-level measurements. Psychol Addict Behav. 2013;27(4):921-33. doi: https://dx.doi.org/10.1037/a0031402. PubMed PMID: 23438241; PubMed Central PMCID: PMCNIHMS481733.

3. Pedersen E, Labrie J. Partying before the party: Examining prepartying behavior among college students. J Am Coll Health. 2007;56(3):237-45. PubMed PMID: 18089504; PubMed Central PMCID: PMCNIHMS63577.

4. Peacock A, Norman T, Bruno R, Pennay A, Droste N, Jenkinson R, et al. Typology of alcohol consumers in five Australian nighttime entertainment districts. Drug and Alcohol Review. 2016;35(5):539-48. doi: https://dx.doi.org/10.1111/dar.12370. PubMed PMID: 26661588.

5. LaBrie J, Pedersen E. Prepartying promotes heightened risk in the college environment: An event-level report. Addict Behav. 2008;33(7):955-9. doi: https://dx.doi.org/10.1016/j.addbeh.2008.02.011. PubMed PMID: 18387749; PubMed Central PMCID: PMCNIHMS52319.

6. Howard A, Patrick M, Maggs J. College student affect and heavy drinking: Variable associations across days, semesters, and people. Psychol Addict Behav. 2015;29(2):430-43. doi: https://dx.doi.org/10.1037/adb0000023. PubMed PMID: 25347017; PubMed Central PMCID: PMCNIHMS636921.

7. Mustonen H, Mäkelä P, Lintonen T. Toward a typology of drinking occasions: Latent classes of an autumn week's drinking occasions. Addiction Research and Theory. 2014;22(6):524-34. doi: 10.3109/16066359.2014.911845.
